# Research progress on endoplasmic reticulum homeostasis in acute kidney injury

**DOI:** 10.3389/fphar.2025.1595845

**Published:** 2025-06-20

**Authors:** Liling Hu, Huike Chen

**Affiliations:** Department of Nephrology, The Second Hospital of Zhuzhou City, Zhuzhou, Hunan, China

**Keywords:** endoplasmic reticulum (ER), ER stress, kidney, AKI, UPR

## Abstract

The endoplasmic reticulum (ER) is the most metabolically active organelle in cells, and recent research has shown that abnormal ER function is involved in the occurrence and development of acute kidney injury (AKI), but the underlying molecular mechanism needs to be further elucidated. Here, we review the biological functions of the ER in cellular metabolism, explore the current research progress on the role of the ER in different triggers of AKI, and summarize the ER stress inhibitors discovered thus far. Finally, we explore the possibility of targeting ER homeostasis as a therapeutic target for AKI.

## 1 Introduction

As an important excretory and regulatory organ in the human body, the normal maintenance of kidney function is crucial for the stability of the internal environment of the body. Acute kidney injury (AKI) is induced when the kidney is severely affected by the external environment and is a common critical illness in clinical practice (Sun et al., 2025). Its main clinical symptom is a sharp decline in renal function in a short period of time, and patients often need urgent medical intervention (Velluto et al., 2025). Previous studies have suggested that inflammation (Ren et al., 2020), mitochondrial dysfunction (Zhao et al., 2021) and oxidative stress (Zhang et al., 2022) are involved in the occurrence and development of AKI. However, these pathogenesis mechanisms cannot fully explain the occurrence of AKI, and drugs developed on the basis of these mechanisms have little effect on clinical treatment. Therefore, it is necessary to explore the mechanism of AKI from other perspectives. Research has shown that abnormalities in endoplasmic reticulum (ER) homeostasis are involved in the occurrence and development of AKI.

The ER is an important organelle in cells and is responsible for key functions such as protein synthesis, folding, modification, and lipid synthesis (Wu et al., 2025). The maintenance of ER homeostasis is highly important for the normal physiological activities of cells. As a highly active metabolic organ, abnormalities in ER homeostasis in the kidney can induce the occurrence and development of AKI (Gallazzini and Pallet, 2018). In AKI, various pathogenic factors such as ischemia, toxicity, infection, and inflammation, can lead to ER stress in intrinsic renal cells (Yan et al., 2018; Ricciardi and Gnudi, 2020). The occurrence of ER stress activates a series of downstream signaling pathways within cells, such as the unfolded protein response (UPR) (Qian et al., 2024), ER-associated degradation (ERAD) (Christianson et al., 2023), and ER-phagy (Hoyer et al., 2024), in an attempt to restore ER homeostasis. When these protective mechanisms are insufficient to cope with sustained stress, ER stress leads to pathological processes such as cell apoptosis, the inflammatory response, and oxidative stress, further exacerbating kidney damage (Chen and Cubillos-Ruiz, 2021; Chen X. et al., 2023; Oakes and Papa, 2015). Studies have shown that drugs or genes that inhibit ER stress can alleviate the occurrence and development of AKI (Liu et al., 2019; Cao et al., 2024; Deng et al., 2023). These findings suggest that targeting ER homeostasis may be a therapeutic target for AKI.

In-depth research on the relationship between ER homeostasis and AKI not only helps us better understand the pathogenesis of AKI but also may provide a theoretical basis for the development of new treatment strategies. This review systematically elucidates the function of the ER and mechanism of ER homeostasis imbalance in AKI, as well as potential therapeutic targets and intervention measures for ER homeostasis regulation, to provide new ideas and directions for the clinical prevention and treatment of AKI.

## 2 The functions of the ER

### 2.1 Ca^2+^ homeostasis

#### 2.1.1 SERCA pumps: uptake and storage

In the cell, the concentrations of calcium ions in different parts of the cell are significantly different; for example, the concentrations of calcium ions in the cytoplasm and the ER can be up to tens of thousands of times different (de Ridder et al., 2023; Dickson et al., 2016). A stable calcium ion concentration is conducive to a variety of cellular processes, such as cell proliferation, differentiation, metabolism, gene transcription and apoptosis (Terrell et al., 2023; Kito and Ohya, 2021; Nicotera et al., 1994). The ER is the main reservoir of calcium ions in the cell, and homeostasis of the ER is critical for maintaining the balance of the intracellular Ca^2+^ concentration. Calcium ions in the ER are mediated by the following proteins: Ca^2+^ pumps, which transport Ca^2+^ from the cytoplasm upward into the ER lumen; Ca^2+^-binding proteins, which bind and store Ca^2+^; and Ca^2+^ channels, which mediate the release of Ca^2+^ from the ER into the cytoplasm (Mekahli et al., 2011). The uptake of calcium ions by the ER is mediated mainly by sarco/ER Ca^2+^ ATPase (SERCA), which belongs to the family of P-type ATPases (Dhureja et al., 2023; Nemirovskaya and Sharlo, 2022). Members of this family also include the plasma membrane Ca^2+^ ATPase, Na^+^/K^+^ ATPase and H^+^/K^+^ ATPase (Sweadner and Donnet, 2001). A feature of these P-type ATPase enzymes is the ability to hydrolyze ATP to ADP while transporting metal ions against the gradient of the SR membrane (Xu and Van Remmen, 2021). The SERCA pump is located on the ER and sarcoplasmic reticulum (SR) and can use the energy generated by ATP hydrolysis to transport Ca^2+^ across membranes. Each SERCA contains two high-affinity Ca^2+^-binding sites, which can transport two Ca^2+^ ions for every ATP molecule hydrolyzed (Wu et al., 2023). SERCA is encoded by three different genes, SERCA1, SERCA2 and SERCA3, and a total of seven different subtypes are expressed in different tissues and cells (Periasamy and Kalyanasundaram, 2007). SERCA pump activity is also regulated by a variety of proteins, such as phospholamban, sarcolipin and myoregulin, which can inhibit SERCA pump activity (Hamstra et al., 2020; Chambers et al., 2022; Rathod et al., 2024), whereas the dwarf open reading frame can effectively activate the SERCA pump (Nelson et al., 2016). The presence of the SERCA pump ensures that the concentration of Ca^2+^ in the ER is much greater than that in the cytoplasm and that high concentrations of Ca^2+^ in the ER are essential for the regulation of posttranslational modification, folding, and protein transport. In the lumen of the ER/SR, Ca^2+^ mainly binds to Ca^2+^ proteins, such as calmodulin in cardiac and skeletal muscle, and calcium-binding proteins such as calnexin or 78-kDa glucose regulatory protein/immunoglobulin heavy chain binding protein (GRP78/BiP), in other tissues (Mekahli et al., 2011).

#### 2.1.2 Ca^2+^ release channels: IP3Rs and RyRs

Inositol 1,4,5-trisphosphate receptors (IP3Rs) and ryanodine receptor (RyR) channels mediate the release of calcium ions from the ER (Ambudkar, 2014). Both have three mammalian isomers, IP3R1, 2, and 3 and RYR1, 2 and 3, which are distributed in different tissues (Luciani et al., 2009; Raturi et al., 2014). IP3R is expressed in most cells, and when IP3 is present, it can bind to IP3R to promote the release of calcium ions from the ER (Zhang et al., 2020). Compared with IP3Rs, RYRs have greater calcium ion binding activity, and the opening of RYRs depends on the concentration of Ca^2+^ in the cytoplasm (Berridge, 2016; Gillespie, 2020).

In general, ER-mediated calcium homeostasis is finely regulated, which ensures various metabolic activities within the cell. The high Ca^2+^ concentration maintained by SERCA is essential for protein folding and posttranslational modifications in the ER lumen.

### 2.2 Protein synthesis and processing

#### 2.2.1 Chaperone-assisted folding and modifications

In cells, the ER is involved in approximately one-third of protein synthesis, folding, and maturation (Anelli and Sitia, 2008). In particular, some proteins on the plasma membrane and organelles are initially translated at ER-bound ribosomes and subsequently transferred to the ER lumen for further processing (Pehar and Puglielli, 2013). The common feature of these proteins is that they have an ER-targeting sequence at the N-terminus, and this signaling sequence is removed when the polypeptide chain is translated. Polypeptide chains entering the ER lumen are folded into unique three-dimensional shapes and undergo various modifications, such as glycosylation, hydroxylation and acylation, in the presence of high calcium ion concentrations and many chaperone proteins (Pehar and Puglielli, 2013; Johnson et al., 2001; Ogawa et al., 2015). These processes ensure that the polypeptide chain is transformed into an active protein, which is then transported to the next step. In the cell, the processes of the folding, transport and degradation pathways of ER proteins are strictly and finely regulated, a process known as ER quality control, which ensures that the synthesized and processed proteins in the ER are conformationally correct and active (Ferro-Novick et al., 2021). ER quality control is achieved by accelerating protein folding, activating the UPR, and eliminating faulty proteins through ERAD (Wiseman et al., 2022).

#### 2.2.2 UPR pathways

In secretory tissues, cells are often in a continuous high-intensity process of protein secretion. For example, islet beta cells synthesize and secrete up to a million insulin molecules per minute (Seino et al., 2011). In the case of insulin resistance, the demand for insulin is greater, which requires islet beta cells to produce more insulin to meet the body’s needs. High-intensity protein secretion is a great challenge for the synthesis and folding of proteins in the ER. When the number of proteins folded far exceeds the upper limit of the capacity of the ER, ER stress often occurs to self-regulate (Di Conza and Ho, 2020; Hetz, 2012). At this point, the UPR detects whether misfolded proteins in the ER have exceeded a threshold. The UPR can regulate the downstream signaling pathway through three different signaling pathways, namely, inositol-requiring enzyme 1α (IRE1α), pancreatic endoplasmic reticulum kinase (PERK) and activating transcription factor 6 (ATF6), thereby reducing the level of ER stress by reducing protein translation and upregulating chaperone expression (Ajoolabady et al., 2023). Unfolded proteins can bind to the lumen domain of Ire1, thereby triggering the self-binding of Ire1 and activating its cytoplasmic effector domain (Credle et al., 2005; Pincus et al., 2010). Proper ER stress effectively regulates the rate of intracellular protein synthesis and maintains cell homeostasis. The persistence of ER stress leads to the continuous activation of the UPR signaling pathway and eventually induces cell death (Marciniak et al., 2022; Rahmati et al., 2018).

#### 2.2.3 ERAD

ERAD is a complex multistep process that involves mainly the recognition, extraction and ubiquitination of ER proteins and ultimately their degradation in the cytoplasmic proteasome (Dreher and Hoppe, 2018). In brief, when the polypeptide chain in the ER cannot be folded, it can be recognized by proteins such as BiP, EDEM, and OS9 in the ER (Sekiya et al., 2017; Seaayfan et al., 2016). The identified substrate is subsequently transported back to the cytoplasm via reverse transcriptional translocation. On the cytoplasmic side of the ER, the substrate is ubiquitinated by ubiquitin ligases and released into the cytoplasm in an ATP-dependent manner, where it is eventually recognized and degraded by the proteasome (Dreher and Hoppe, 2018; Blackwood et al., 2023). ERAD ensures that the unfolded protein is cleared in time, thus maintaining protein homeostasis in the cell.

In addition to protein processing, the ER is also the primary site for lipid metabolism, where synthesized lipids are stored in droplets or transported via membrane contact sites.

### 2.3 Lipid synthesis and droplet biogenesis

In the cell, the ER is also the key site of lipid metabolism and synthesis and contains many lipid synthetases, such as DGAT1/2 and phosphatidylserine synthase (PSS) (Farese and Walther, 2023; Wang and Benning, 2012). Lipid droplets store neutral fat in the cell, and they are also considered to constitute a single layer of phospholipid membrane organelles (Zadoorian et al., 2023). A previous review adequately described the role of the ER in lipid droplet formation (Walther et al., 2017). In addition, the ER is involved in lipid synthesis along with other organelles. For example, there are mitochondria-associated membranes (MAMs) between the ER and mitochondria, and MAMs are involved in the synthesis of phospholipids and cholesterol (Vance, 2015; Vance, 2014). The part of the ER connected to the Golgi apparatus is rich in tubules and vesicles called the ER-Golgi intermediate compartment (ERGIC), which is involved in the synthesis and redistribution of phospholipids in cells (Schwarz and Blower, 2016).

### 2.4 ER-phagy: Selective autophagy of the ER

ER-phagy is a newly discovered type of selective autophagy in which the ER can directly bind to LC3 through ER-phagy receptors, thereby mediating ER degradation. ER-phagy maintains the homeostasis of the ER and normal cellular function by clearing damaged, redundant, or dysfunctional components of the ER (Gonzalez et al., 2023). Currently, multiple ER proteins, such as FAM134B, SEC62, reticulon-3 (RTN3), cell cycle progression 1 (CCPG1), atlastin-3 (ATL3), and TEX264, have been shown to mediate the occurrence of ER-phagy, (Gubas and Dikic, 2022; Chino and Mizushima, 2023). When ER homeostasis is abnormal, ER-phagy receptors bind with LC3 to induce dysfunctional ER degradation, thereby blocking secondary cellular dysfunction (Stolz and Grumati, 2019; Chino and Mizushima, 2020).

In cells, the UPR, ERAD and ER-phagy precisely assist each other in jointly maintaining ER homeostasis. When stimulated by the outside world, unfolded and misfolded proteins accumulate in cells. At this time, the early warning system (UPR) in the cell is activated to reduce the number of misfolded proteins through promoting the expression of chaperone proteins and protein-folding enzymes, inhibiting the transcription and translation of proteins, strengthening the degradation tool (ERAD), etc. When the persistent UPR fails to restore the homeostasis of the ER in time, cells activate ER-phagy to degrade the functionally impaired ER. Therefore, the UPR, ERAD, and ER-phagy together form a refined collaborative network that maintains ER homeostasis in cells. They do not operate independently but form a hierarchical defense system through dynamic interactions at the temporal, spatial, and molecular levels, in which they respond to ER stress.

## 3 ER homeostasis and AKI

**FIGURE 1:**
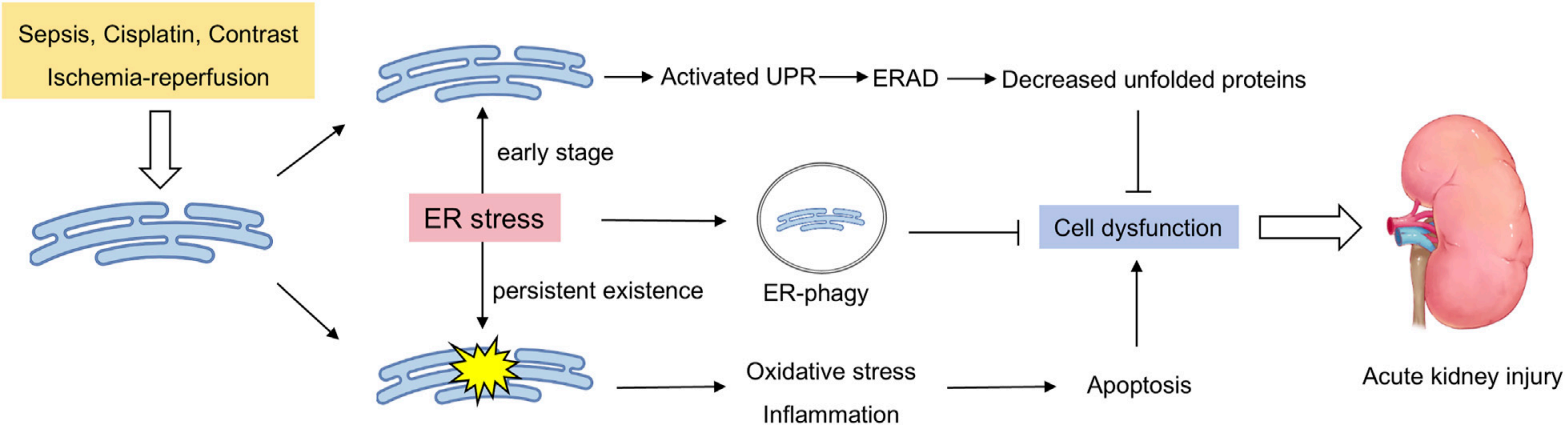
Double-sided endoplasmic reticulum stress in acute renal injury. External stimuli induce ER stress in the kidneys, which activates the unfolded protein response to clear unfolded proteins in a timely manner. Moreover, the occurrence of ER-phagy ensures that the damaged ER is cleared in a timely manner. When these protective mechanisms are insufficient to cope with sustained stress, ER stress leads to pathological processes such as cell apoptosis, the inflammatory response, and oxidative stress, further exacerbating kidney damage.

With increasing research revealing the importance of the ER in maintaining kidney function, the relationships between abnormal ER homeostasis and the occurrence and development of AKI have also been explored (Figure 1). In the following section, we summarize the current research progress on ER homeostasis abnormalities in AKI induced by different etiologies.

### 3.1 Cisplatin-induced AKI

Cisplatin is a very effective chemotherapy drug that is often used to treat solid tumors. Nephrotoxicity is the main side effect, and the risk of nephrotoxicity in cisplatin-treated patients is between 20% and 35%, manifested mainly as severe renal tubule injury and acute renal failure (Fang et al., 2021). In the kidney, cisplatin can be passively absorbed into renal tubule cells by organic cation transporter 2 (OCT2) and then continuously accumulate in the kidney (Eljack et al., 2014). The entry of cisplatin into the urine is mediated by transporters such as multidrug resistance-associated proteins (MRPs) and multiantimicrobial extrusion proteins (MATEs) (Holditch et al., 2019). Severe nephrotoxicity severely limits the use of cisplatin. At present, there is still a lack of effective drugs to prevent cisplatin-induced AKI. There are many molecular mechanisms of cisplatin-induced AKI, such as oxidative stress (Huang et al., 2017) and inflammation (Yu et al., 2023; Chen et al., 2019). However, with the increasing research, the role of ER homeostasis in cisplatin-induced AKI progression has also been revealed.

Multiple studies have shown that ER stress is overactivated in the kidneys of individuals with cisplatin-induced AKI and that the inhibition of ER stress can effectively slow kidney injury (Yang et al., 2024; Liu et al., 2024). NSC228155 is a novel compound with anticancer and antibacterial effects, and it reduces the ER stress level of cisplatin-induced renal tubular cells and HK-2 cells and inhibits apoptosis (Li et al., 2022). Similar results revealed that *achyranthes aspera* extract and dexmedetomidine attenuate cisplatin-induced kidney damage by inhibiting ER stress (Lin et al., 2024; Chai et al., 2020). Moreover, disturbances in energy metabolism are present in cisplatin-induced AKI and exacerbate the progression of AKI. Deceased fatty acid oxidation (FAO) levels, inhibited ATP production and increased lipid deposition in the kidneys of patients with cisplatin-induced AKI, while increasing the level of FAO effectively protected the kidney function (Xu et al., 2022; Li et al., 2020). The main site of fatty acid oxidation is the mitochondria; however, the MAM domain is located between the ER and mitochondria, and the ER can regulate mitochondrial function through the MAM (Senft and Ronai, 2015; Doghman-Bouguerra and Lalli, 2019). The MAM mediates the flow of calcium ions from the ER into the mitochondria, and increased levels of calcium ions promote mitochondrial ATP synthesis (Sun and Ding, 2020; Li et al., 2019; Barazzuol et al., 2021). In addition, markers of ER-mediated cell death, such as caspase-12 and calpain, are activated in rat kidney tissue (Peyrou et al., 2007). Although a number of studies have revealed that overactivation of ER stress in cisplatin-induced AKI and that ER stress inhibitors can effectively reverse renal injury, how ER homeostasis is disrupted in cisplatin-induced AKI, and its molecular mechanism have yet to be determined. These mechanisms need to be further explored in future studies to better develop drugs that target ER stress.

### 3.2 Ischemia‒reperfusion injury (IRI)

The kidney is a very sensitive organ to ischemia and hypoxia. Renal ischemia‒reperfusion injury is a common complication after transplantation and heart surgery. Renal blood circulation is restricted during kidney transplantation, which is indispensable for kidney reperfusion after surgery, resulting in kidney damage (Zhao et al., 2020). Kidney cells change from aerobic respiration to anaerobic respiration when the oxygen supply of the kidney decreases, the production of ATP in the kidney also decreases, and the accumulation of lactic acid increases (Nieuwenhuijs-Moeke et al., 2020). Moreover, a decrease in calcium ion excretion leads an increase in its intracellular concentration. When reperfusion occurs, the recovery of the oxygen content leads to the production of many reactive oxygen species, and the concentration of intracellular calcium ions further increases, inducing cell death (Nieuwenhuijs-Moeke et al., 2020). In addition, ER dysfunction plays a key role in the pathophysiological process of renal ischemia-reperfusion injury.

During IRI, renal cells undergo hypoxia, oxidative stress, and disruption of calcium homeostasis, all of which can lead to ER stress. Multiple studies have shown that renal IRI induces ER stress in renal tubular epithelial cells. Tang et al. demonstrated through single-cell RNA sequencing that kidney cells from ischemic AKI patients who differentially expressed genes in renal proximal tubular cells were enriched mainly in ER stress signals (Tang et al., 2023). Similar evidence has been reported in the kidneys of both mice and rats (Zhang et al., 2020; Tang et al., 2020), and persistent ER stress exacerbates kidney damage, whereas drug-mediated inhibition of ER stress can effectively slow kidney damage (Zhang et al., 2024). However, the role of ER stress in IRI still needs further exploration. Although most studies have shown that inhibiting ER stress can alleviate IRI-related renal damage, the role of the ER is different in the early stages of the disease. Chandrika et al. reported that the use of the ER stress inducer tunicamycin to intervene in renal tubular epithelial cells activated ER stress, increased the expression of the ER chaperone protein Grp78, and triggered downstream autophagy pathways, thereby inhibiting the activation of caspase-3 and cell death (Chandrika et al., 2015). This means that when ER stress is in the early to middle stages, autophagy can be induced to promptly clear damaged proteins and organelles, whereas when ER stress is too severe or excessive, the apoptotic pathway may be activated. Therefore, when the ER is targeted as a preventive and therapeutic target for renal IRI, controlling and monitoring ER stress levels is crucial.

### 3.3 Sepsis

Sepsis is a serious form of organ dysfunction caused mainly by the host’s dysregulated response to infection (Srzic et al., 2022). When faced with severe infections, the body produces excessive inflammatory factors such as interleukins and tumor necrosis factor, and experiences endothelial damage and abnormal secretion of vasoactive substances, which can trigger AKI (He et al., 2022). Bagshaw et al. reported that approximately 64.4% of septic shock patients develop early AKI (Bagshaw et al., 2009). However, even in patients without severe sepsis or shock, AKI is still common: 34% of nonsevere community-acquired pneumonia patients develop AKI (Murugan et al., 2010). Regardless of the species, disease stage, and severity of sepsis, three pathophysiological changes are consistently observed in sepsis patients and animal models: microcirculation dysfunction, inflammation, and the bioenergetic adaptive response to injury (Zarbock et al., 2014). Overall, the occurrence of sepsis AKI significantly increases the risk of in-hospital mortality (2-6 times) and is closely associated with the likelihood of later progression to chronic kidney disease (Hoste et al., 2015; Pais et al., 2024). However, there is still a lack of specific molecular markers and treatment methods for sepsis-induced AKI in clinical practice. Therefore, a deeper understanding of the pathogenesis of sepsis-induced AKI is necessary. Recently, multiple studies have shown that abnormal ER homeostasis is involved in the occurrence and development of sepsis-induced AKI.

Excessive misfolded proteins in the ER can further exacerbate ER stress and lead to apoptosis. Molecular chaperones are particularly important for promoting protein folding in the ER. Porter et al. demonstrated that knocking out the ER-resident protein GRP170 resulted in an acute kidney injury phenotype in mice (Porter et al., 2022). Moreover, in a cell model induced by lipopolysaccharide (LPS), the expression of the key protein GRP78 in ER stress is elevated, accompanied by an increase in apoptosis. The absence of GRP78 can alleviate the LPS-induced immune response and oxidative stress (Teng et al., 2018). Similar results have also been reported, with a significant increase in ER stress marker proteins in septic AKI mouse or rat models (Sun et al., 2024; Luo et al., 2020). Drug or gene knockout-mediated inhibition of ER stress can effectively delay sepsis-induced AKI-related kidney damage. Sun et al. reported that Marins-1 treatment significantly inhibited kidney damage in AKI model mice induced by cecal ligation and puncture, whereas an AMPK inhibitor (Compound C) partially blocked the protective effect of Marins-1 (Sun et al., 2024). Although multiple studies have revealed that maintaining ER homeostasis and inhibiting ER stress can effectively alleviate sepsis-induced AKI, current research has several limitations. At present, the molecular mechanism of ER stress in sepsis-induced AKI is not clear. Currently, only ER stress has been observed during sepsis-induced AKI, but its molecular mechanism still needs to be explored. Therefore, a deeper understanding of the molecular mechanism of ER stress in the occurrence of sepsis AKI is beneficial for targeting ER homeostasis as a therapeutic drug for sepsis AKI in the future.

### 3.4 Contrast-induced (CI) AKI

CI-AKI refers to the sudden deterioration of renal function caused by intravenous or arterial injection of iodinated contrast agents (Mccullough et al., 2016). Its main manifestation is a sudden and long-term decline in renal function that occurs 48–72 h after injection (Ward and Valentovic, 2019). It was first described by Bartels et al., in 1954 (Bartels et al., 1954). With the development of follow-up imaging, the incidence rate of CI-AKI has gradually increased. Although the incidence of CI-AKI is low in the general population, it is significantly greater in high-risk groups, such as those with renal insufficiency, diabetes, dehydration, heart failure and elderly individuals (Rundback et al., 2011). Currently, there is still a lack of effective prevention and control measures for CI-AKI. Recent studies suggest that ER dysfunction may be involved in the occurrence and development of CI-AKI. The radiocontrast agent meglumine diatrizoate can upregulate the expression of GRP78, ATF4, and CHOP to induce ER stress, leading to the activation of the renin‒angiotensin system and the apoptosis of renal tubular cells in rats. However, pretreatment with valsartan significantly inhibited ER stress levels and renal injury (Sun et al., 2017). Apelin is an endogenous antioxidant and anti-inflammatory physiological regulator (Vinel et al., 2018). Liu et al. reported that exogenous apelin-13 can alleviate cell and renal tissue damage in rats induced by contrast agent intervention by inhibiting ER stress (Liu et al., 2023). These studies have partially revealed the role of ER stress in CI-AKI, and the inhibition of ER stress can alleviate CI-AKI injury. However, the molecular mechanism of ER stress activation in CI-AKI needs to be further explored.

## 4 ER stress inhibitors and clinical translation

Given the role of ER stress in the occurrence and development of different types of AKI, targeting ER stress is a potential approach for developing drugs for the prevention and treatment of AKI in the future. Currently, multiple studies have reported that some compounds or drugs can alleviate AKI by inhibiting ER stress, and we have summarized these finding here (Table 1).

**TABLE 1:** ER stress inhibitors.

Type of compounds	Name	Target	Diseases	References
ER-targeted agents	GSK2606414	PERK/p-eIF2α/ATF4/CHOP axis	Cerebral ischemia Periodontitis	Zhou et al. (2023), Gao et al. (2024)
	Ceapins	ATF6α	Cancer	Gallagher et al. (2016)
	AA147	ATF6	Multiple sclerosis	Aksu et al. (2025)
	ISRIB	eIF2B	Traumatic brain injury, Breast cancer	Zhou et al. (2024), Lee et al. (2022)
	Salubrinal	eIF2α	Insulin resistance Breast cancer, Cholangiocarcinoma	Nguyen et al. (2024), Alsterda et al. (2021), Yu et al. (2018)
	KIRA6	IRE1α	Cancer Traumatic brain injury	Luo et al. (2024), Shi et al. (2023)
Chemical Compounds	Dexmedetomidine	PERK IRE1 ATF6 GRP78	Cisplatin-induced AKI, Traumatic brain injury	Chai et al. (2020), Sun et al. (2020)
	Recombinant human erythropoietin	CHOP GRP78	Cisplatin-induced AKI, Nonalcoholic fatty liver disease	Kong et al. (2013), Hong et al. (2019)
	TUG891	ATF6, PERK, eIF2α, XBP1	Cisplatin-induced AKI, Intraventricular hemorrhage	(Huang et al., 2020; Wang et al., 2023)
	Melatonin	GRP78, IRE1, PERK, ATF4	Nonalcoholic fatty liver disease Periodontitis	Guan et al. (2023), Cui et al. (2023)
	Valsartan	eiF2α, CHOP, Grp78, ATF-4	Doxorubicin-induced cardiotoxicity	Wu et al. (2023), Kim et al. (2022)
	Pioglitazone	IRE1α, Xbp1, Grp78	Diabetes Cardiovascular disease	Hong et al. (2018), Soliman et al. (2019)
	Exendin-4	PERK/CHOP/eiF2α axis, ATF-4, Xbp1	Diabetes	Oh et al. (2013)
Natural compounds	Resveratrol	eiF2α, CHOP, GRP78, Xbp1	Cancer	Rojas et al. (2014)
	Forsythiaside A	GRP78, PERK, CHOP, ATF4	Septic acute liver injury, Sepsis-induced AKI	Fu et al. (2023), Chen X. et al. (2023)
	Pinocembrin	ATF4, eiF2α	Sepsis-induced AKI	Zhang et al. (2022)
	Leonurine	ATF4, CHOP	Cisplatin-induced AKI	Zhang et al. (2022)
	Naringenin	GRP78, CHOP	Renal ischemia‒ reperfusion	Zhang et al. (2022)
	Puerarin	IRE1α, PERK, eIF2α	Acute myocardial infarction	Xue et al. (2024)

Drug intervention targeting ER homeostasis is a promising approach for the treatment of AKI. At present, small molecule inhibitors of key proteins in the ER stress pathway and some chemical chaperones, such as 4-PBA, have been found to improve AKI by inhibiting ER stress in cell and animal models, but there are still challenges that need to be further addressed in the use of ER stress as a target for the treatment of AKI in the future. First, many ER stress regulators lack specificity and may have unexpected effects on other cellular processes. For example, some regulators may reduce ER stress while inhibiting the necessary UPR pathways required for cell survival. In addition, ER stress has both protective and harmful effects. Excessive inhibition may hinder necessary adaptive responses, whereas excessive activation may lead to apoptosis and inflammation. Therefore, how to precisely regulate ER stress to achieve the best balance between adaptation and cell death is a considerable challenge. Most of the existing preclinical models use acute injury conditions and cannot fully represent the complexity of human AKI, especially in the case of chronic kidney injury. Furthermore, there may be differences in ER stress responses between animal models and human tissues, making it difficult for preclinical research results to accurately predict clinical outcomes and limiting the transformation of research results into human applications. Finally, at present, relatively few studies on ER stress in samples from AKI patients exist. The very limited clinical research data make it difficult to determine the exact role and therapeutic effect of ER stress in human AKI, and evaluations of the safety and efficacy of related drugs in patients with kidney diseases are lacking.

## 5 Conclusion

AKI is a common critical disease with high morbidity and mortality. There is an urgent need to investigate its pathogenesis and find effective treatments. The role of ER homeostasis in acute renal injury has been thoroughly investigated. While the findings were surprising, there were several limitations. The double-edged sword effect and spatiotemporal dynamics of ER stress in AKI are worthy of exploration. ER stress is a protective response in the early stage that aims to restore ER homeostasis. However, persistent or excessive ER stress results in proapoptotic and proinflammatory responses. How to precisely define this “turning point” and the exact role (whether it is a driving factor or an accompanying phenomenon) of ER stress at different stages of AKI remain unclear. Moreover, there is complex crosstalk and feedback regulation among the three main pathways of the UPR (PERK, IRE1α, and ATF6). In AKI, how these pathways precisely coordinate to determine cellular outcomes is not fully understood. Currently, research on ER stress and AKI often uses animal models, such as rats and mice. However, there are differences between animal models and humans in terms of physiology, pathology, and immune response, which limits the applicability of research results in humans. In the future, research should focus on the precise regulation of the ER stress signaling pathway and the development of safe and effective treatment strategies. Overall, targeting ER homeostasis is an effective potential therapeutic target for AKI.
